# Human papillomavirus awareness and vaccination willingness among adults in Madagascar: a cross-sectional study

**DOI:** 10.1186/s12905-025-04199-9

**Published:** 2025-12-03

**Authors:** Aaron Remkes, Alexina Zafinimampera, Olivette Totofotsy, Fesia Elveric Ratiaharison, Haingoniavo Garcia Rambeloson, Elly Daus, Myriam Lassmann, Alexandra Schmidt, Fiona Franz, Pia Rausche, Jana Hey, Tiana Randrianarisoa, Tahinamandranto Rasamoelina, Irina Kislaya, Diavolana Andrianarimanana, Jürgen May, Valentina Marchese, Rivo Andry Rakotoarivelo, Daniela Fusco

**Affiliations:** 1 Implementation research, Bernhard Nocht Institute for Tropical Medicine, Hamburg, Germany; 2 German Center for Infection Research, Hamburg-Borstel-Lübeck-Riems, Germany; 3 Faculty of Medicine, University of Fianarantsoa, Fianarantsoa, Madagascar; 4 Centre d’Infectiologie Charles Mérieux (CICM), University of Antananarivo, Antananarivo, 101 Madagascar; 5 Department of Infectious Diseases Epidemiology, Bernhard Nocht Institute for Tropical Medicine, Hamburg, Germany; 6 Centre Hospitalier Universitaire (CHU), Androva, Madagascar

**Keywords:** Human papillomavirus, Vaccination programmes, Health literacy, Madagascar, Vaccination willingness, Disease awareness

## Abstract

Disease awareness is key to utilising healthcare services, including vaccinations. Madagascar, with high human papillomavirus (HPV) prevalence (> 36%), marked cervical cancer rates, and low general routine vaccination coverage, faces major challenges in managing HPV-related diseases, mostly because of its fragile health system and the absence of HPV vaccination programmes. This study aimed to assess HPV awareness and vaccination willingness in Madagascar to inform strategies for implementing HPV vaccination programmes in the country. A cross-sectional survey among adults was conducted in the Boeny and Matsiatra Ambony regions. HPV awareness and willingness to be vaccinated against HPV were analysed using Poisson regression. Overall, the level of HPV awareness was low (4.6%; 95% CI: 3.8–5.6). Urban residency (aPR = 1.7; 95% CI: 1.1–2.6), higher education (aPR = 2.1; 95% CI: 1.1-4.0), and recent contact with healthcare services (aPR = 1.8; 95% CI: 1.2–2.6) were associated with greater HPV awareness. HPV vaccination willingness was generally high (67.0%; 95% CI: 65.0–69.0) but was significantly higher among participants aware of HPV (aPR = 1.4; 95% CI: 1.2–1.5). Most participants received ample information on general routine vaccinations, with healthcare workers (HCWs) reported as the most reliable source of information (48.6%). This study shows that improving knowledge about HPV could increase the success of HPV vaccination programmes in the country. In addition, the study highlights the importance of education in terms of health literacy, stressing that efforts to reach rural populations with lower levels of education are crucial.

**Supplementary Information:**

The online version contains supplementary material available at 10.1186/s12905-025-04199-9.

## Background

Awareness and knowledge of diseases are essential components of health literacy (HL) and indispensable for utilising healthcare services (HCSs) [1, 2]. HL plays a critical role in disease management and prevention, for example in the context of vaccinations, which are among the most effective measures for reducing the health consequences of infections such as those caused by the human papillomavirus (HPV) [2–5]. Furthermore, awareness and knowledge of HPV have been shown to be positively associated with HPV vaccination willingness [1, 2, 6, 7].

HPV is the most common sexually transmitted infection worldwide; it can also be transmitted through skin contact, and chronic infection can cause various cancers, most notably cervical cancer (CC) [8–10]. In 2022, the World Health Organisation (WHO) reported 620,000 cancer deaths in women and 70,000 in men worldwide related to carcinogenic HPV strains, of which 350,000 were due to CC [10, 11]. Among these cases, more than 90% occurred in low- and middle-income countries (LMICs) [12]. HPV prevalence among women is highest in sub-Saharan Africa (SSA), at approximately 24%, which is double the global average of around 12% [9, 11]. In SSA, CC accounts for the majority of cancer-related mortality, disproportionately affecting the most marginalised population groups [12–14].

Vaccination against HPV is one of the most cost-effective interventions for the prevention of CC, especially in low-resource settings or underserved populations where cancer treatment and access to comprehensive HCSs are not widely available [15–17]. Following the introduction of the first HPV vaccine in 2006, CC incidence, morbidity and mortality have decreased in most countries that achieved high vaccination coverage [18–20], whereas in resource-limited areas and LMICs with low vaccination coverage or lacking vaccination programmes, CC rates have stagnated or continued to rise [13, 14, 21].

In August 2020, the World Health Organization adopted the global strategy for CC elimination, including the 90-70-90 strategy to eliminate CC as a public health problem in all member states by 2120, with the main goals being to fully vaccinate 90% of girls aged 9–14, screen 70% of women first at age 35 and again at age 45 for CC, and treat 90% of women with diagnosed CC by 2030 [22]. This objective does not include HPV vaccination for boys, even though it has already been introduced by several high-income countries (HICs), as males can also develop HPV-related diseases and cancers and contribute to the transmission cycle [23, 24] in the context of low vaccination among girls. Nevertheless, global HPV vaccination coverage is low, with only 31% of girls having at least started the vaccination regime [25]. To date, 158 out of 194 WHO member states (≈ 81%) have already included HPV vaccination in their national immunisation programmes, however only 32 of the 47 countries in SSA (≈ 68%) have done so [25].

In Madagascar, the prevalence of HPV is relatively high compared to other countries in SSA and, according to the few studies available, lies at over 36% [26, 27]. CC is the most common cancer and accounts for almost 20% of all reported cancer cases in the country [17, 28]. Nonetheless, preventive measures for HPV and CC are scarce: reportedly, only 8% of women have ever been screened for CC [17]. Apart from a pilot project between 2013 and 2015, vaccination against HPV has not yet been implemented in the country, let alone included in the national vaccination programme [17, 29]. Furthermore, Madagascar exhibits one of the lowest overall routine vaccination rates in the world [30, 31]. The availability and accessibility of the highly centralised healthcare system is a major obstacle in Madagascar, especially regarding the uptake of vaccinations [31–33]. More than one-third of communities (35.3%) are located more than 10 km away from the nearest HCS [34]. In 2023, Madagascar had only approximately four healthcare workers (HCWs - defined as individuals providing HCSs within the health system) per 10,000 inhabitants available, with healthcare provision in rural communities, where 60% of the population resides, being even more precarious [33–35]. In addition to the availability and accessibility of HCSs, HL, especially awareness and knowledge of diseases, has been shown to play a critical role in the adoption of health measures among Malagasy populations [36–38].

This study aims to assess awareness of HPV as well as willingness to vaccinate against HPV to inform implementers on how to build HPV vaccination programmes in Madagascar. The study also investigated the perceived reliability of information sources and the amount of information available about general routine vaccines and vaccination programmes in the country. The study was conducted in two regions of Madagascar, Boeny and Matsiatra Ambony, where no previous HPV immunisation pilots were conducted [29].

## Methods

### Study design, setting and inclusion criteria

A cross-sectional survey was implemented within two regions of Madagascar: Matsiatra Ambony in the Province of Fianarantsoa in the central highlands and Boeny in the Province of Mahajanga on the northwest coast of Madagascar, with estimated populations of 1,444,587 and 929,312, respectively [39]. In Boeny, three urban Fokontany (the smallest basic administrative subdivisions at the commune level in Madagascar) were included: Fiofio (−15.715508, 46.3262379), Mangarivotra (−15.7164838, 46.3134356), and Tsaramandroso Ambony (−15.7107543, 46.3169778), along with three rural Fokontany, namely Ankazomborona (−16.115517, 46.7524297), Antanimora (−16.095893, 46.688823), and Mangapaika (−15.961847, 46.686315). In Matsiatra Ambony, three urban Fokontany were included: Ivory (−21.447184, 47.092717), Talatamaty (−21.4364479,47.1058769), Rova (−21.461164, 47.075342), and three rural Fokontany, namely, Alakamisy Ambohimaha (−21.3216683, 47.2253729), Talata Iboaka (−21.343732, 47.182600), and Tambohivo Ambohimaha (−21.298946, 47.249276). These regions and study sites were selected to reflect diverse sociodemographic, cultural and economic contexts, high estimated HPV prevalence [26, 27], and the absence of prior HPV-related pilot studies [29], allowing assessment of HPV awareness, knowledge and vaccination willingness across diverse Malagasy populations.

## Sample size and participant selection strategy

The sample was stratified by region and level of urbanisation. The minimum required sample size was determined to estimate an expected prevalence of 50% with an absolute precision of ± 5% and a confidence level of 95%. In each stratum, at least 480 participants were targeted, with more populous Fokontany being assigned proportionally more participants.

Within each Fokontany, 6–12 door-to-door routes were designed, starting at randomly generated geographic coordinates within the geographic boundaries of the Fokontany using Lemeshow and Robinson’s algorithm [40]. Households were selected systematically, with one person per household invited to participate. In order to balance the distribution of the sample by sex and age group, interviewers selected each subsequent participant based on the characteristics of the previously recruited individual, following a structured algorithm: (a) select the oldest available woman; (b) select the oldest available man; (c) select any available man; (d) select any available woman.

Participants who met the following inclusion criteria were included in the study: (i) were older than 18 years, (ii) were fluent in French and/or Malagasy, and (iii) provided voluntary written informed consent. A total of 2,145 participants were initially recruited. Following quality assessment by the data management team, six participants were excluded because they did not meet the plausibility checks or because of missing responses in critical study variables, including HPV awareness, sex, or age.

## Study instrument

An individual questionnaire (Supplementary File 1) was employed by the study’s interviewers and completed based on participants’ responses to collect data on their sociodemographic characteristics (including biological sex rather than gender, due to biological sex-specific differences in HPV infection and related outcomes), as well as participants’ awareness of HPV, knowledge of HPV transmission routes, and understanding of the recommended vaccination age. However, knowledge of the link between HPV infection and CC was not assessed. Additionally, all the participants were asked about their willingness to receive an HPV vaccine, as well as their willingness to recommend the vaccine to others and to have their children vaccinated against HPV. General information about where and how to obtain reliable information about vaccines and vaccination programmes were also gathered through a multiple-choice questionnaire given to participants. Following the HPV awareness questions, interviewers were directed to provide a concise account of HPV, its associated consequences, and the available vaccine to the participants who were previously unaware of HPV before they were asked about their willingness to be vaccinated if a vaccine was offered. The interviewers were instructed not to prompt answers. The survey was created by adapting existing validated tools on the basis of previously used questionnaires from similar research studies [37, 38]. The questionnaire items were formulated through detailed consultations with the local principal investigator and the research team to ensure that they were relevant and appropriate for the context. Additional validation was achieved after piloting and adapting the questionnaire following the training of the interviewers. Data collectors were trained by experienced healthcare workers and research staff from Germany and Madagascar in good clinical practice, ethical principles, electronic data capture, survey procedures, and HPV knowledge, with a pilot survey ensuring proficiency and standardisation before field deployment. The Malagasy version of the questionnaire was back translated into French and validated by the principal investigator.

## Data collection and management

Data collection took place between 18 August and 19 October 2023 in the 12 Fokontany previously described. All participants were asked to sign a consent form before being interviewed by two trained interviewers from each region in French or Malagasy at the randomly selected coordinates.

A participant identifier was given to all participants to ensure data protection through pseudonymisation. All answers were initially recorded on a paper-based questionnaire that was then transcribed by double data entry and merged by a data manager in an electronic case report form (eCRF) using REDCap^®^ (Vanderbilt University, Nashville, USA) hosted on a server located at Bernhard Nocht Institute for Tropical Medicine, Hamburg, Germany. The completed eCRFs were monitored live on a daily basis and subjected to quality assessment and validation following standard operating procedures to ensure accurate data processing.

### Statistical analysis

Numerical variables are presented using medians and interquartile ranges (IQRs) if non-parametric and means and standard deviations (SDs) if parametric. Categorical variables are presented as frequencies and percentages. Owing to missing values resulting from participant omissions or implausible/erroneous answers excluded, the denominator may differ in some calculations. Awareness of HPV was described through prevalence and the exact (Clopper-Pearson) 95% confidence interval (95% CI). Awareness was defined when a participant stated that they had heard about HPV prior to their participation in the survey. Knowledge regarding HPV transmission and vaccination was only assessed in a subsample of participants aware of HPV. Willingness was defined at three levels by asking participants how likely they were to have themselves and/or their children vaccinated against HPV, with their responses categorised as willing or hesitant.

For each outcome, crude and adjusted prevalence ratios (cPRs and aPRs) with 95% CIs were estimated using Poisson regression with robust standard errors. This method was preferred over logistic regression because, in cross-sectional studies, it allows direct estimation of PRs and is less prone to convergence issues compared to the log-binomial model [41]. These models were employed for HPV awareness and willingness to receive HPV vaccination as outcome variables, with appropriate adjustments made for potential confounding variables. Model fit was assessed using residual deviance, variance inflation factors (VIF) were computed to evaluate multicollinearity. All the statistical analyses and illustrations were performed using R software version 4.2.2 [42].

## Results

### Characteristics of the study participants

As depicted in Table 1, the study included a total of 2,139 study participants, with a relatively even distribution across the Boeny (48.2%) and Matsiatra Ambony (51.8%) regions, urban (49.5%) and rural (50.5%) Fokontany, and female (59.0%) and male (41.0%) participants. The median age of the participants was 30 years (IQR = 23–45), ranging from 18 to 86 years. The largest age group was between 20 and 29 years of age (37.7%). Most participants reported being Christians (85.8%), having completed at least secondary school (69.1%), working (76.3%), and having both female and male children (40.8%). With respect to healthcare access, fewer than half of the participants reported contact with HCSs within the last year (45.3%). The mean distance to the nearest PHC facility was reported to be 29.5 min (SD = 26.4). The vast majority (82.1%) of respondents reported that the next PHC facility was within one hour’s reach.

**Table 1:** Characteristics of study participants

	*n* (%)
Region (*n* = 2,139)	
Boeny	1,031 (48.2%)
Matsiatra Ambony	1,108 (51.8%)
Urbanicity (*n*= 2,139)	
Rural	1,081 (50.5%)
Urban	1,058 (49.5%)
Sex (*n*= 2,139)	
Female	1,261 (59.0%)
Male	878 (41.0%)
Age in years	
Median [IQR]	30 [23–45]
Age group (in years) (*n*= 2,139)	
18–19	257 (12.0%)
20–29	807 (37.7%)
30–39	370 (17.3%)
≥ 40	705 (33.0%)
Religion (*n*= 2,135*)	
Christian	1,835 (85.9%)
Non-Christian	300 (14.1%)
Highest level of education (*n*= 2,138*)	
No/primary school	660 (30.9%)
Secondary school	1,053 (49.2%)
Higher education	425 (19.9%)
Employment status (*n*= 2,132*)	
Working	1,632 (76.6%)
Retired/unemployed	163 (7.6%)
Student	337 (15.8%)
Children (*n*= 2,126*)	
Female child(ren) only	277 (13.0%)
Male child(ren) only	324 (15.2%)
Female and male children	872 (41.1%)
No child(ren)	653 (30.7%)
Contact to HCS within the last year (*n*= 2,133*)	
No	1,163 (54.5%)
Yes	970 (45.5%)
Distance to nearest PHC facility (*n*= 2,120*)	
≤ 60 min	1,757 (82.9%)
≥ 60 min	363 (17.1%)
Distance to nearest PHC facility in minutes (*n*= 2,120*)	
Mean [SD]	29.5 [26.4]

Sample sizes (n) and proportions (%) of participants per category, along with standard deviations (SDs) and interquartile ranges (IQRs), where relevant, are reported. An asterisk (*) indicates deviations in sample sizes from the total number of 2,139 participants due to missing data.

## HPV awareness and associated factors

**Fig. 1:**
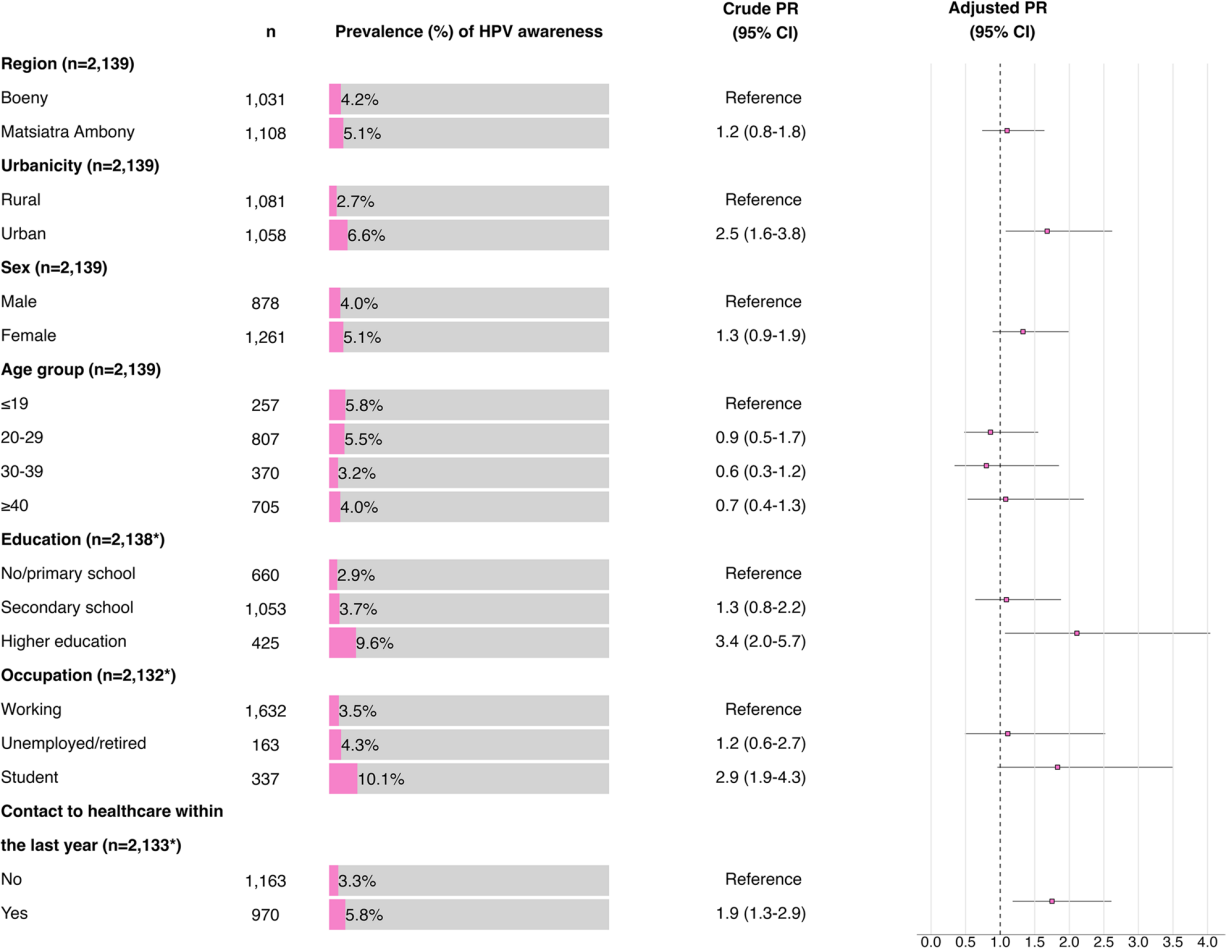
Prevalence and crude and adjusted prevalence ratios (PRs) for HPV awareness, adjusted for region, urbanicity, sex, age group, education, occupation, and contact with HCSs, based on Poisson regression analysis. The 95% confidence intervals (CIs) and sample sizes (*n*) are reported. An asterisk (*) indicates deviations in sample sizes from the total of 2,139 participants due to missing data. Model fit: deviance = 553.9, df= 2,113, *p* ≈ 1.000; 1.03

A total of 99 out of 2,139 participants (4.6%; 95% CI: 3.8–5.6) reported having heard of HPV prior to this survey (see Supplementary Table S2). The prevalence of HPV awareness was comparable between the regions of Boeny (4.2%; 95% CI: 3.0–5.6.0.6) and Matsiatra Ambony (5.1%; 95% CI: 3.8–6.5) (Fig. 1 and Supplementary Table S2). However, participants from urban Fokontany presented higher levels of HPV awareness than did those from rural Fokontany (6.6%; 95% CI: 5.2–8.3 vs. 2.7%; 95% CI: 1.8–3.8). HPV awareness was similar between female and male participants, with 5.1% (95% CI: 3.9–6.4) and 4.0% (95% CI: 2.8–5.5), respectively. Among the age groups, individuals aged between 18 and 19 years (5.8%; 95% CI: 3.3–9.4) presented the highest HPV awareness, whereas those aged 30–39 years presented the lowest level, at 3.2% (95% CI: 1.7–5.6). The participants with higher education reported the highest level of HPV awareness at 9.6% (95% CI: 7.0–12.9.0.9), followed by those who completed secondary school at 3.7% (95% CI: 2.6–5.0.6.0), whereas those with no or primary school education reported the lowest level of awareness at 2.9% (95% CI: 1.7–4.5). Students displayed the highest level of HPV awareness across all categories, with a value of 10.1% (95% CI: 7.1–13.8). Participants who reported contact to HCSs in the past year demonstrated a higher level of HPV awareness (6.3%; 95% CI: 4.8–8.0) compared with those without such contact (3.3%; 95% CI: 2.3–4.5).

Figure 1 shows that the HPV awareness was significantly greater among individuals from urban Fokontany (aPR = 1.7; 95% CI: 1.1–2.6), those with a higher level of education (aPR = 2.1; 95% CI: 1.1–4.0.1.0), and individuals who reported contact with the HCSs in the past year (aPR = 1.8; 95% CI: 1.2–2.6). No significant associations were observed for other variables after adjusting for confounders.

### Knowledge about HPV

Among the 99 participants who reported being aware of HPV, 72.4% reported knowing the route of transmission of HPV, of which 81.7% stated that the correct route of transmission was through sexual contact, but only 8.5% reported that it was through skin contact (see Supplementary Table S1). Transmission through contact with infected blood was the most frequently reported incorrect route of transmission at 19.7%. Among the HPV-aware participants, the majority (64.6%) considered an age of over l to be suitable for HPV vaccination. In contrast, 34.4% considered the age group of 9–17 years to be suitable, which includes the 9–14 year range recommended by the WHO [9]. Among those 99 participants, 74.7% reported perceiving the consequences of HPV as very serious or extremely serious, whereas 13.1% reported perceiving the consequences of HPV as moderately serious and 7.1% as slightly to not serious at all.

### Vaccination willingness in relation to HPV awareness

As shown in Supplementary Table S3, 67.0% (95% CI: 65.0–69.0) of the participants stated that they were willing to be vaccinated against HPV. Vaccination willingness was greater among the participants who were aware of HPV than those who were not aware (84.8% (95% CI: 76.5–90.6) vs. 66.1% (95% CI: 64.0–68.2.0.2)).

As shown in Supplementary Table S4, 71.7% (95% CI: 69.7–73.6) of participants reported a willingness to have their daughter vaccinated, and as shown in Supplementary Table S5, 71.1% (95% CI: 69.1–73.0) reported a willingness to have their son vaccinated against HPV. Among participants who reported being aware of HPV, 82.7% (95% CI: 74.0–88.9.0.9) were willing to have their daughter and 81.2% (95% CI: 72.3–87.8) were willing to have their son vaccinated. Among those who were not aware of HPV, the corresponding figures were 71.2% (95% CI: 69.2–73.1) and 70.6% (95% CI: 68.6–72.6), respectively.

**Fig. 2:**
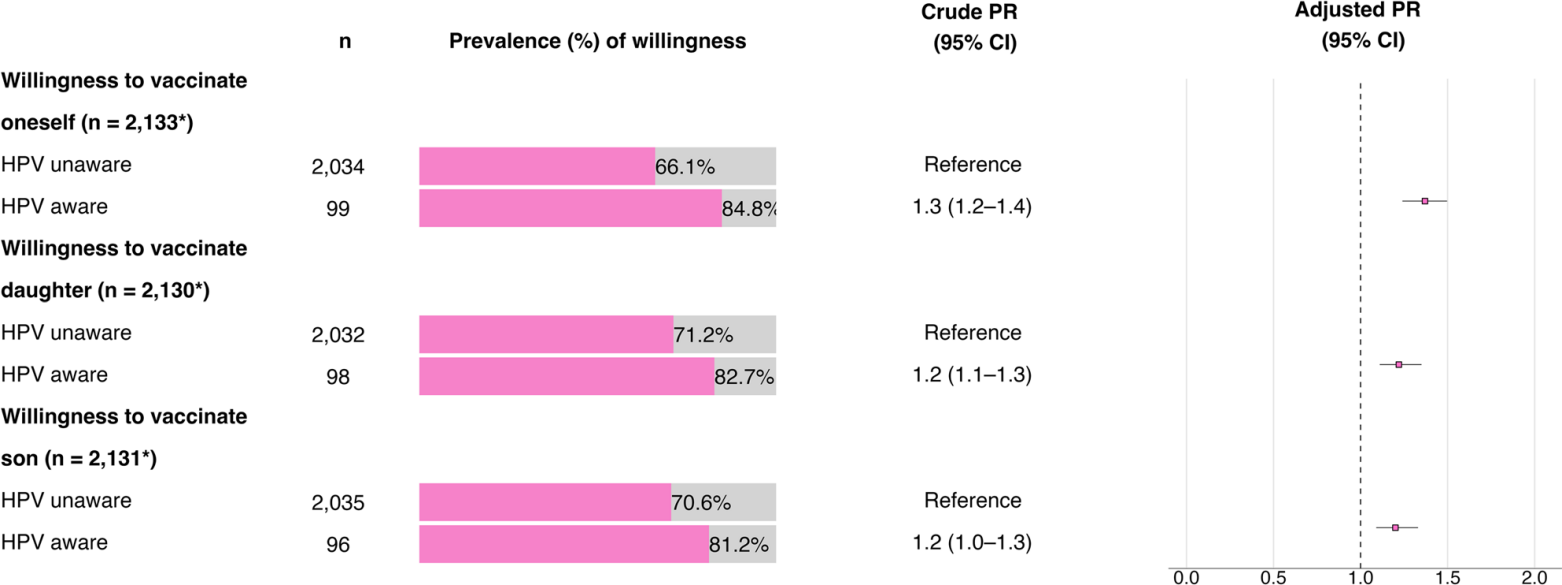
Prevalence and crude and adjusted prevalence ratios (PRs) for willingness to vaccinate oneself, one's daughter, and one's son, in relation to HPV awareness, adjusted for region, urbanicity, sex, age group, education, occupation, and contact with HCSs, based on Poisson regression analysis. 95% confidence intervals (CIs) and sample sizes (*n*) are reported. An asterisk (*) indicates deviations in sample sizes from the total of 2,139 participants due to missing data. Model fit for willingness to vaccinate a) oneself: deviance = 1,075.8, df = 2,107, *p* ≈ 1.000; 1.03<VIF<1.77; b) daughter: deviance = 956.99, df = 2,105, *p* ≈ 1.000; 1.03<VIF<1.82; c) son: deviance 977.4, df = 2,108, *p* ≈ 1.000; 1.03<VIF<1.82

As can be seen in Fig. 2 (or Supplementary Table S3-S5), the aPR was significantly greater for the participants aware of HPV than for the participants who were not aware of it in terms of willingness to be vaccinated against HPV (aPR = 1.4; 95% CI: 1.2–1.5) or to have their daughter (aPR = 1.2; 95% CI: 1.1–1.4) or son (aPR = 1.2; 95% CI: 1.1–1.3) vaccinated.

Detailed data on HPV vaccination willingness for oneself, one’s daughter, and one’s son according to sociodemographic variables are provided in Supplementary Tables S3–S5.

### Information on vaccines and vaccination programmes

Regarding general information on vaccines or vaccination programmes, 77.5% of all participants indicated knowing where to find accurate and up-to-date information on this matter, whereas only 22.3% reported not knowing.

**Fig. 3:**
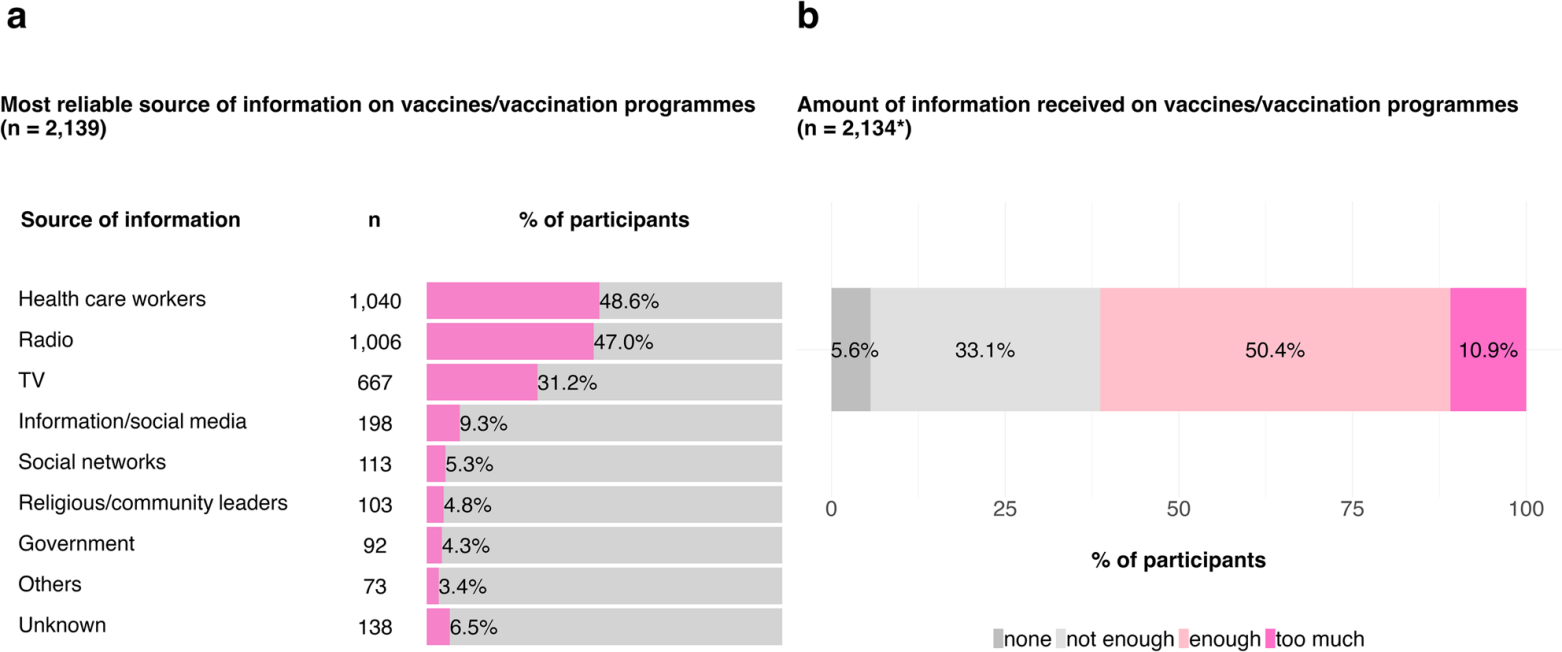
Proportion (%) of participants’ responses to (**a**) the most reliable sources of information on vaccines and vaccination programmes, and (**b**) the amount of information received. Panel **a** shows the distribution of participants' responses (multiple choice) regarding which sources they consider most reliable for information about vaccines and vaccination programmes. Panel **b** illustrates the proportion of participants who reported receiving different amounts of information about vaccines and vaccination programmes, ranging from “none” to “too much”. Sample sizes (*n*) are reported. An asterisk (*) indicates deviations in sample sizes from the total of 2,139 participants due to missing data

As depicted in Fig. 3 A, HCW (48.6%), radio (47.0%), and television (31.2%) were commonly reported to be the most reliable sources of information on vaccines and/or vaccination programmes, whereas online information or social media (9.3%), social networks such as friends, family, and neighbours (5.3%), religious or community leaders (4.8%), the government (4.3%), or other sources of information, including the workplace, university, school, celebrities, or home visits (3.4%), were less frequently reported as reliable sources of information. Moreover, 6.5% of the respondents indicated uncertainty regarding what the most reliable source of information might be. Figure 3B shows that the majority of participants reported receiving enough (50.4%) or too much information (10.9%) on vaccines and/or vaccination programmes, whereas 33.0% reported not receiving enough information, and only 5.6% reported not receiving any information.

Among the participants who were aware of HPV, 28.3% reported having heard or seen information regarding HPV vaccination prior to this survey, whereas 70.7% had never heard or seen it (see *Supplementary Table S1*). After this survey, 76.3% of these HPV-aware participants stated that they were “extremely likely”, and 18.6% said they were “quite likely” to recommend the vaccine to others. However, 5.2% indicated that they did not recommend the vaccine or were uncertain.

## Discussion

This study reported very low HPV awareness (4.6%; 95% CI: 3.8–5.6) in two regions of Madagascar where no immunisation programmes against HPV infection have been implemented to date [17]. In August 2024, the Malagasy Ministry of Health formally engaged with the Global Alliance for Vaccines and Immunisation (GAVI, the Vaccine Alliance) to start the importation of HPV vaccines into the country, with the objective of launching immunisation programmes by the end of 2025. The findings of this study will contribute to the design of awareness-raising initiatives that support implementation strategies and high vaccination coverage.

Among the factors influencing HPV awareness, higher education is positively associated, with participants more than twice as likely to be aware of it (aPR = 2.1; 95% CI: 1.1–4.0.1.0). Not surprisingly, as seen in similar studies performed both in the SSA region [43, 44] and in HICs [1, 7, 45], higher education is commonly associated with higher levels of awareness of diseases. However, health promotion information is frequently disseminated using informative posters, flyers and booklets, which often miss individuals as well as whole populations with lower levels of education [46]. In addition, effective education strategies for those groups that provide information about HPV and its preventative measures are under-researched in SSA [47], and the language used for disseminating health messages is frequently too complex and not always context-adapted, leading to lower interest from people with lower levels of education [46].

The second factor positively associated with awareness of HPV identified was recent contact with HCSs (aPR = 1.8; 95% CI: 1.2–2.6). This suggests that information and knowledge about diseases are well disseminated in healthcare settings and that individuals are more likely to pay attention and attribute higher reliability to health education information in such a context, as has also been shown in similar studies conducted in SSA countries [45, 48]. Residence in an urban area was also identified as a positive factor associated with HPV awareness (aPR = 1.7; 95% CI: 1.1–2.6). This emphasises the importance of health education in rural areas, which are generally already underserved in terms of the accessibility and availability of HCSs, especially visible in terms of the general routine vaccination coverage rate there [30, 31, 33, 34]. In addition, rural areas are often disproportionately affected by disease, as is the case for HPV in Madagascar [26]. The literature describes the female sex and gender as a positive factor for HPV awareness [1, 49]. However, this study did not replicate any association between HPV awareness and sex. Evidence suggests that women in Madagascar face greater challenges in accessing health information, resulting in lower awareness, particularly regarding diseases that predominantly affect women [38, 50].

Given the low level of HPV awareness, our study could only provide an exploratory description of knowledge about HPV. The proportion of participants who knew the correct vaccination age recommended by the WHO was relatively low (34.3%). Although the majority identified HPV as an STI (81.7%), only a small minority (8.5%) cited skin contact as a transmission route. It must be emphasised that HPV awareness does not necessarily equate to accurate knowledge, such as about adequate preventive measures [51]. False, alleged knowledge based on misinformation hinders the willingness to vaccinate, as the HPV vaccination pilot project in Madagascar has shown, due to the stigmatisation associated with the sexually transmitted nature of the disease [29].

Vaccine hesitancy and willingness are dynamic features that change depending on the vaccine, disease, context and time [52]. It has been shown that knowledge about infectious diseases can in some cases reduce risk perception and thus prevent the adoption of preventive measures [53, 54]. However, with respect to HPV in Madagascar, most participants perceived the personal consequences of infection as very to extremely serious during the survey (74.7%) and therefore perceived a high level of risk that has been shown to positively influence an individual’s vaccination decision [55, 56].

Among the participants who reported being aware of HPV, only 28.3% had already received information about HPV vaccination prior to this study. On the other hand, when asked about general information on vaccines and implemented vaccination programmes, the participants reported that they had received enough (50.4%) or even too much (10.9%) information in the past. It is important to note that this questions referred to vaccinations and vaccination programmes in general, highlighting that, despite participants perceiving themselves as well-informed, both general routine vaccine coverage [31] and HPV awareness remain low. Although awareness and knowledge about HPV generally increase vaccination willingness and uptake [1, 2, 6, 7], this study shows how important it is to design communication strategies that promote and strengthen HL without causing detrimental effects, delivering targeted, clear information that distinguishes HPV vaccines from routine immunisations and avoids information fatigue, as could be seen with the so-called infodemic in the context of the COVID-19 pandemic [57].

To improve awareness and knowledge about HPV, it is important to understand the means of communication favoured by the target populations [1, 48]. Previous studies have emphasised HCSs and the radio as important sources of information in LMICs [45, 48]. In our study, HCWs (48.6%) and radio (47.0%) were also the most frequently mentioned most reliable sources of information, with television (31.2%) being the third most frequently mentioned source. Online or social media, religious or community leaders, family, friends or neighbours, and the government are rarely mentioned as reliable sources, which has been highlighted in similar contexts before but not to this extent [48, 58].

Surprisingly, the willingness to have one’s daughter and/or son vaccinated was comparable (71.7% (CI: 69.7–73.6) vs. 71.1% (CI: 69.1–73.0)), whereas previous studies in SSA reported a reluctance of parents to vaccinate their sons, especially in countries where the vaccine has already been introduced [59, 60]. This element should be monitored and further explored. While the WHO recommendations target girls for HPV vaccination [9], studies have shown the potential to increase immunisation coverage when gender-neutral strategies are adopted [61].

Despite the strengths of this study, which are reflected in the context-adapted research instrument, the probabilistic sampling and the diversity of participants, it is not without limitations. Given the nature of our study, we cannot rule out social desirability bias. The cross-sectional survey design limits causal conclusions. Willingness to be vaccinated was assessed among participants who reported no prior knowledge of HPV. For this reason, given the small number of HPV-aware participants, vaccination willingness was not reported in the main text stratified by sociodemographic (e.g., parenthood, sex), as such subgroup analysis could yield misleading interpretations in a largely unaware population; these data are provided in *Supplementary Tables S3-S5*. Further, only participants aged 18 or older were included, meaning that our results cannot reflect HPV awareness or vaccination willingness in the target age group for HPV vaccination. Even though some general information about the virus was given after the awareness question, more structured knowledge of the disease could cause a shift in willingness depending on how the severity and relevance would be reassessed. The high level of unawareness of HPV has led to a limitation in analysing knowledge due to the resulting limited sample size. Additionally, deep knowledge regarding the consequences of chronic HPV infections such as CC remains unexplored.

In conclusion, this study provides indications that can inform specific recommendations for roll-out strategies once the vaccine becomes available in the country. Specifically, improving knowledge about HPV could increase the success of vaccination programmes in the country. In addition, the study makes clear that efforts to reach rural populations with lower levels of education are crucial. Working with community HCWs could increase the spread and uptake of awareness campaigns, as they are the most trusted source of information. Given the limited availability and accessibility of HCSs in Madagascar, outreach to community HCWs could have a positive effect on awareness and ultimately vaccination coverage and uptake.

## Supplementary Information

Supplementary Material 1.

Supplementary Material 2.

Supplementary Material 3.

Supplementary Material 4.

Supplementary Material 5.

Supplementary Material 6.

## Data Availability

The datasets generated during and/or analysed during the current study are available from the corresponding author upon reasonable request.
