# Supplementary material for: Human papillomavirus awareness and vaccination willingness among adults in Madagascar: a cross-sectional study
Source: BMC Womens Health. 2025 Dec 3;25:596. doi: 10.1186/s12905-025-04199-9 (PMC12706918; doi:10.1186/s12905-025-04199-9)
Supplement: Supplementary file 1 — Supplementary Material 1. [file 12905_2025_4199_MOESM1_ESM.docx]

**HPV Survey Questionnaire (English Translation)**

**I. IDENTIFICATION**

**I01. Date of the interview (DD-MM-YYYY):**

**I02. Study Municipality:**

**I03. Respondent PID:**

**I04. Are inclusion criteria satisfied (>18 years)?**

- Yes
- No

**I05. Was informed consent obtained?**

- Yes
- No

**I06. Interviewer’s name:**

**I07. Interview start time (24h system):**

**I08. Interview end time (24h system):**

**I09. Interview administrative region:**

- Boeny
- Fianarantsoa

**I09.1. If Boeny, which Fokontany:**

- Ankazomborona
- Antanimora
- Mangapaika
- Fiofio
- Mangarivotra
- Tsaramandroso Ambony

**I09.2. If Fianarantsoa, which Fokontany:**

- Ivory
- Talatamaty
- Rova
- Talata Iboaka
- Alakamisy Ambohimaha
- Tambohivo Ambohimaha

**A. RESPONDENT BACKGROUND**

**A01. How old are you?**
(If not exact, ask for best estimate)
Age in years: ________

**A02. What is your sex?**

- Female
- Male

**A03. Do you have living children?**

- Yes, daughter(s)
- Yes, son(s)
- Yes, both
- No

**A04. Please select the age groups of your children (check all that apply):**

- 0–8 years
- 9–14 years
- 15–17 years
- 18+ years

**A05. What are your MAIN languages of communication in your daily life?**

- French
- Malagasy
- Local language
- No preference

**A05.1. If local language, please specify:**

**A06. What is your main religion?**

- Christian (e.g., Catholic, Evangelical, Jehovah’s Witness, other Christian sect)
- Muslim
- Traditional religion
- Other

**A06.1. If other, please specify:**

**A07. Have you ever attended school?**

- Yes
- No

**A08. What is the highest level of education you have reached?**

- Primary school or less
- Secondary school
- University
- Vocational training
- Other

**A08.1. If other, please specify:**

**A09. What is your employment status?**

- Unemployed
- Student / Apprentice
- Employee
- Self-employed
- Other

**A09.1. If other, please specify:**

**A10. What is your main occupation?**

- Homemaker
- Driver
- Worker
- Seller
- Agriculture / Livestock / Fishing
- Teacher
- Health worker
- Other

**A10.1. If other, please specify:**

**A11. Have you consulted a healthcare professional (doctor, nurse, midwife, community health worker) in the past 12 months?**

- Yes
- No
- Don’t know

**A12. If you are sick, how long (on average) does it take to reach the health center (CSB)?** HH:MM ________

**B. HPV & VACCINATION EXPERIENCE**

**B01. Have you ever heard of HPV (human papillomavirus)?**

- Yes
- No
- Don’t know

**B02. Do you know how HPV is transmitted?**

- Yes
- No

**B03. How is HPV transmitted? (Select all that apply)**

- Contaminated water
- Breathing contaminated air
- Drinking contaminated water
- Fishing in contaminated water
- Eating unwashed food
- Washing dishes/laundry in river/lake
- Poor hygiene/sanitation
- Poor genital hygiene
- Swimming in river/lake
- Working in rice fields/open farming
- Contact with infected blood
- Sexual contact
- Skin contact
- Shared food/utensils
- Urinating in contaminated area
- Not transmitted/spontaneous
- Other
- Don’t know

**B03.1. If other, please specify:**

**B04. Have you ever been screened for HPV?**

- Yes
- No
- Don’t know

**B05. Have you ever tested positive for HPV?**

- Yes
- No
- Don’t know

**B14. How worried are you about being infected with HPV?**

- Extremely worried
- Very worried
- Somewhat worried
- A little worried
- Not worried at all
- Don’t know

**B15. How worried are you that your son might contract HPV?**

- Extremely worried
- Very worried
- Somewhat worried
- A little worried
- Not worried at all
- Don’t know
- I don’t have a son

**B16. How worried are you that your daughter might contract HPV?**

- Extremely worried
- Very worried
- Somewhat worried
- A little worried
- Not worried at all
- Don’t know
- I don’t have a daughter

**C. SOCIAL NORMS AND DECISION TO VACCINATE AGAINST HPV**

**C01. Have you ever heard or seen information about HPV vaccination?**

- Yes
- No
- Don’t know

**C02. Are community leaders (religious, political, teachers, health professionals) in your community supportive of HPV vaccines?**

- Yes
- No
- Don’t know

**C03. If yes, please specify which leaders (check all that apply):**

- Religious leaders
- Political leaders
- Teachers
- Health workers
- Other
- Don’t know

**C03.1. If other, please specify:**

**C04. Do you think most people at your workplace or school would get vaccinated against HPV if recommended?**

- Yes
- No
- Don’t know

**C05. Do you think most of your friends and family members would get vaccinated against HPV if recommended?**

- Yes
- No
- Don’t know

**C06. According to you, which age groups should be vaccinated against HPV? (Select all that apply)**

- Infants (0–2)
- Children (3–8)
- Adolescents (9–17)
- Adults (18+)
- Don’t know

**C07. What is your perception of the consequences of HPV?**

- Not serious at all
- Slightly serious
- Moderately serious
- Very serious
- Extremely serious
- Don’t know

**C08. If you had access to an HPV vaccine, would you want to be vaccinated?**

- Definitely yes
- Probably yes
- Probably no
- Definitely no
- Don’t know

**C09. If you had a daughter, would you agree for her to be vaccinated if an HPV vaccine were offered?**

- Yes, definitely
- Probably yes
- Probably no
- No, definitely not
- Don’t know

**C10. If you had a son, would you agree for him to be vaccinated if an HPV vaccine were offered?**

- Yes, definitely
- Probably yes
- Probably no
- No, definitely not
- Don’t know

**D. INFORMATION ABOUT VACCINATION**

**D01. What are the most reliable sources of information about vaccines or vaccination programs? (Select all that apply)**

- Government
- Workplace / Employer
- School / University
- Health workers
- Radio
- TV
- News media (print or online)
- Social media
- Religious leader
- Community leaders
- Neighbors / Family / Friends
- Artists / Musicians
- Other
- Don’t know

**D01.1. If other, please specify:**

**D02. What do you think about the amount of information you receive about vaccines or vaccination programs?**

- I don’t receive any information
- I don’t receive enough information
- I receive sufficient information
- I receive too much information

**D03. Do you know where to find accurate and up-to-date information on vaccines or vaccination programs?**

- Yes
- No
- Don’t know
