# Supplementary material for: Human papillomavirus awareness and vaccination willingness among adults in Madagascar: a cross-sectional study
Source: BMC Womens Health. 2025 Dec 3;25:596. doi: 10.1186/s12905-025-04199-9 (PMC12706918; doi:10.1186/s12905-025-04199-9)
Supplement: Supplementary file 2 — Supplementary Material 2. [file 12905_2025_4199_MOESM2_ESM.docx]

**Supplementary Table S1.** Sample sizes (n) and proportions (%) of participants’ knowledge about HPV and the HPV vaccination.

|  | **n (%)** |
| --- | --- |
| **Awareness about HPV transmission (n = 98*)** |  |
| Yes | 71 (72.4) |
| No | 27 (27.6) |
| **Reported HPV transmission route (n = 71*)** |  |
| Sexual contact | 58 (81.7) |
| Skin contact | 6 (8.5) |
| Contact with contaminated blood | 14 (19.7) |
| Contact with contaminated water | 5 (7.0) |
| Breathe contaminated air | 6 (8.5) |
| Others | 2 (2.8) |
| **Perceived severity of the consequences of HPV (n = 99)** |  |
| Not serious at all | 2 (2.0) |
| Slightly serious | 5 (5.1) |
| Moderately serious | 13 (13.1) |
| Very serious | 53 (53.5) |
| Extremely serious | 21 (21.2) |
| Uncertain | 5 (5.1) |
| **Recommended age group for HPV vaccination (n = 99)** |  |
| 0-2 years | 6 (6.1) |
| 3-8 years | 8 (8.1) |
| 9-17 years | 34 (34.4) |
| ≥18 years | 64 (64.6) |
| Unknown | 12 (12.1) |
| **Received any information about the HPV vaccination (n = 99)** |  |
| Yes | 28 (28.3) |
| No | 70 (70.7) |
| Uncertain | 1 (1.0) |
| **Would recommend HPV vaccine to others after the survey (n = 97*)** |  |
| Extremely likely | 74 (76.3) |
| Quite likely | 18 (18.6) |
| Not at all likely | 3 (3.1) |
| Uncertain | 2 (2.1) |

An asterisk (*) indicates deviations in sample sizes from the total number of 99 HPV-aware participants due to missing data.
