# Supplementary material for: Human papillomavirus awareness and vaccination willingness among adults in Madagascar: a cross-sectional study
Source: BMC Womens Health. 2025 Dec 3;25:596. doi: 10.1186/s12905-025-04199-9 (PMC12706918; doi:10.1186/s12905-025-04199-9)
Supplement: Supplementary file 3 — Supplementary Material 3. [file 12905_2025_4199_MOESM3_ESM.docx]

**Supplementary Table S2.** Prevalence, crude (cPR) and adjusted prevalence ratios (aPR) for HPV awareness, adjusted for HPV awareness, sociodemographic and healthcare-related factors (Poisson regression analysis).

|  | **n** | **Prevalence % (95% CI)** | **Crude PR (95% CI)** | **Adjusted PR (95% CI)** |
| --- | --- | --- | --- | --- |
| **Total (n = 2,139)** | 99 | 4.6 (3.8-5.6) | - | - |
| **Region (n = 2,139)** |  |  |  |  |
| Boeny | 1,031 | 4.2 (3.0-5.6) | Reference | Reference |
| Matsiatra Ambony | 1,108 | 5.1 (3.8-6.5) | 1.2 (0.8-1.8) | 1.1 (0.7-1.6) |
| **Urbanicity (n = 2,139)** |  |  |  |  |
| Rural | 1,081 | 2.7 (1.8-3.8) | Reference | Reference |
| Urban | 1,058 | 6.6 (5.2-8.3) | 2.5 (1.6-3.8) | 1.7 (1.1-2.6) |
| **Sex (n = 2,139)** |  |  |  |  |
| Male | 878 | 4.0 (2.8-5.5) | Reference | Reference |
| Female | 1,261 | 5.1 (3.9-6.4) | 1.3 (0.9-1.9) | 1.3 (0.9-2.0) |
| **Age group (n = 2,139)** |  |  |  |  |
| 18-19 | 257 | 5.8 (3.3-9.4) | Reference | Reference |
| 20-29 | 807 | 5.5 (4.0-7.3) | 0.9 (0.5-1.7) | 0.9 (0.5-1.6) |
| 30-39 | 370 | 3.2 (1.7-5.6) | 0.6 (0.3-1.2) | 0.8 (0.3-1.9) |
| ≥40 | 705 | 4.0 (2.7-5.7) | 0.7 (0.4-1.3) | 1.1 (0.5-2.2) |
| **Highest level of education**  **(n = 2,138*)** |  |  |  |  |
| No/primary school | 660 | 2.9 (1.7-4.5) | Reference | Reference |
| Secondary school | 1,053 | 3.7 (2.6-5.0) | 1.3 (0.8-2.2) | 1.1 (0.6-1.9) |
| Higher education | 425 | 9.6 (7.0-12.9) | 3.4 (2.0-5.7) | 2.1 (1.1-4.0) |
| **Employment status (n = 2,132*)** |  |  |  |  |
| Working | 1,632 | 3.5 (2.7-4.5) | Reference | Reference |
| Unemployed/retired | 163 | 4.3 (1.7-8.6) | 1.2 (0.6-2.7) | 1.1 (0.5-2.5) |
| Student | 337 | 10.1 (7.1-13.8) | 2.9 (1.9-4.3) | 1.8 (1.0-3.5) |
| **Contact to healthcare within the last year (n = 2,133*)** |  |  |  |  |
| No | 1,163 | 3.3 (2.3-4.5) | Reference | Reference |
| Yes | 970 | 6.3 (4.8-8.0) | 1.9 (1.3-2.9) | 1.8 (1.2-2.6) |

95% Confidence intervals (CI) and sample sizes (n) are provided. An asterisk (*) indicates deviations in sample sizes from the total number of 2,139 participants due to missing data.
