# Supplementary material for: Human papillomavirus awareness and vaccination willingness among adults in Madagascar: a cross-sectional study
Source: BMC Womens Health. 2025 Dec 3;25:596. doi: 10.1186/s12905-025-04199-9 (PMC12706918; doi:10.1186/s12905-025-04199-9)
Supplement: Supplementary file 4 — Supplementary Material 4. [file 12905_2025_4199_MOESM4_ESM.docx]

**Supplementary Table S3.** Prevalence, crude (cPR) and adjusted prevalence ratios (aPR) for HPV vaccination willingness for oneself, adjusted for HPV awareness, sociodemographic and healthcare-related factors (Poisson regression analysis).

|  | **n** | **Prevalence % (95% CI)** | **Crude PR**  **(95% CI)** | **Adjusted PR (95% CI)** |
| --- | --- | --- | --- | --- |
| **Total** | 2,133 | 67.0 (65.0-69.0) | - | - |
| **HPV awareness (n = 2,133*)** |  |  |  |  |
| HPV unaware | 2,034 | 66.1 (64.0-68.2) | Reference | Reference |
| HPV aware | 99 | 84.8 (76.5-90.6) | 1.3 (1.2–1.4) | 1.4 (1.2–1.5) |
| **Region (n = 2,133*)** |  |  |  |  |
| Boeny | 1,031 | 77.6 (74.9-80.0) | Reference | Reference |
| Matsiatra Ambony | 1,102 | 57.1 (54.1-60.0) | 0.7 (0.7–0.8) | 0.7 (0.7–0.8) |
| **Urbanicity (n = 2,133*)** |  |  |  |  |
| Rural | 1,079 | 71.7 (69.0–74.3) | Reference | Reference |
| Urban | 1,054 | 62.1 (59.2–65.0) | 0.9 (0.8–0.9) | 0.9 (0.8–0.9) |
| **Sex (n = 2,133*)** |  |  |  |  |
| Male | 877 | 68.2 (65.0–71.2) | Reference | Reference |
| Female | 1,256 | 66.2 (63.5–68.7) | 1.0 (0.9–1.0) | 1.0 (0.9–1.0) |
| **Age group (n = 2,133*)** |  |  |  |  |
| 18-19 | 256 | 71.9 (66.1–77.0) | Reference | Reference |
| 20-29 | 804 | 69.3 (66.0–72.4) | 1.0 (0.9–1.1) | 1.0 (0.9–1.1) |
| 30-39 | 369 | 66.7 (61.7–71.3) | 0.9 (0.8–1.0) | 0.9 (0.8–1.0) |
| ≥40 | 704 | 62.8 (59.2–66.3) | 0.9 (0.8–1.0) | 0.8 (0.8–0.9) |
| **Education (n = 2,133*)** |  |  |  |  |
| No/primary school | 658 | 70.8 (67.2–74.2) | Reference | Reference |
| Secondary school | 1,052 | 67.1 (64.2–69.9) | 0.9 (0.9–1.0) | 1.0 (0.9–1.0) |
| Higher education | 423 | 60.8 (56.0–65.3) | 0.9 (0.8–0.9) | 0.9 (0.8–1.0) |
| **Occupation (n = 2,126*)** |  |  |  |  |
| Working | 1,628 | 67.8 (65.5–70.0) | Reference | Reference |
| Unemployed/retired | 163 | 67.5 (60.0–74.2) | 1.0 (0.9–1.1) | 0.9 (0.8–1.1) |
| Student | 335 | 63.9 (58.6–68.8) | 0.9 (0.9–1.0) | 0.9 (0.8–1.0) |
| **Contact to healthcare**  **within the last year**  **(n = 2,127*)** |  |  |  |  |
| No | 1,161 | 65.8 (63.0–68.5) | Reference | Reference |
| Yes | 966 | 68.6 (65.6–71.5) | 1.0 (1.0–1.1) | 1.1 (1.0–1.2) |

95% Confidence intervals (CI) and sample sizes (n) are provided. An asterisk (*) indicates deviations in sample sizes from the total number of 2,139 participants due to missing data.
