# Supplementary material for: Human papillomavirus awareness and vaccination willingness among adults in Madagascar: a cross-sectional study
Source: BMC Womens Health. 2025 Dec 3;25:596. doi: 10.1186/s12905-025-04199-9 (PMC12706918; doi:10.1186/s12905-025-04199-9)
Supplement: Supplementary file 5 — Supplementary Material 5. [file 12905_2025_4199_MOESM5_ESM.docx]

**Supplementary Table S4.** Prevalence, crude (cPR) and adjusted prevalence ratios (aPR) for HPV vaccination willingness for one’s daughter, adjusted for HPV awareness, sociodemographic and healthcare-related factors (Poisson regression analysis).

|  | **n** | **Prevalence % (95% CI)** | **Crude PR**  **(95% CI)** | **Adjusted PR (95% CI)** |
| --- | --- | --- | --- | --- |
| **Total** | 2,130 | 71.7 (69.7-73.6) | - | - |
| **HPV awareness (n = 2,130*)** |  |  |  |  |
| HPV unaware | 2,032 | 71.2 (69.2–73.1) | Reference | Reference |
| HPV aware | 98 | 82.7 (74.0–88.9) | 1.2 (1.1–1.3) | 1.2 (1.1–1.3) |
| **Region (n = 2,130*)** |  |  |  |  |
| Boeny | 1,031 | 82.0 (79.5–84.2) | Reference | Reference |
| Matsiatra Ambony | 1,099 | 62.1 (59.2–64.9) | 0.8 (0.7–0.8) | 0.8 (0.7–0.8) |
| **Urbanicity (n = 2,130*)** |  |  |  |  |
| Rural | 1,080 | 75.8 (73.2–78.3) | Reference | Reference |
| Urban | 1,050 | 67.4 (64.5–70.2) | 0.9 (0.8–0.9) | 0.9 (0.9–1.0) |
| **Sex (n = 2,130*)** |  |  |  |  |
| Male | 877 | 71.9 (68.8–74.7) | Reference | Reference |
| Female | 1,256 | 71.6 (69.0–74.0) | 1.0 (0.9–1.1) | 1.0 (0.9–1.0) |
| **Age group (n = 2,130*)** |  |  |  |  |
| 18-19 | 254 | 78.0 (72.5–82.6) | Reference | Reference |
| 20-29 | 803 | 74.5 (71.3–77.4) | 1.0 (0.9–1.0) | 1.0 (0.9–1.1) |
| 30-39 | 370 | 68.4 (63.5–72.9) | 0.9 (0.8–1.0) | 0.9 (0.8–1.0) |
| ≥40 | 703 | 68.0 (64.5–71.3) | 0.9 (0.8–0.9) | 0.9 (0.8–0.9) |
| **Education (n = 2,129*)** |  |  |  |  |
| No/primary school | 658 | 77.6 (74.2–80.6) | Reference | Reference |
| Secondary school | 1,052 | 71.1 (68.3–73.8) | 0.9 (0.9–1.0) | 0.9 (0.9–1.0) |
| Higher education | 423 | 63.9 (59.2–68.3) | 0.8 (0.8–0.9) | 0.8 (0.7–0.9) |
| **Occupation (n = 2,125*)** |  |  |  |  |
| Working | 1,630 | 71.7 (69.4–73.8) | Reference | Reference |
| Unemployed/retired | 162 | 74.1 (66.8–80.2) | 1.0 (0.9–1.1) | 1.0 (0.9–1.1) |
| Student | 333 | 71.5 (66.4–76.1) | 1.0 (0.9–1.1) | 1.0 (1.0–1.2) |
| **Contact to healthcare**  **within the last year**  **(n = 2,124*)** |  |  |  |  |
| No | 1,159 | 70.8 (68.1–73.3) | Reference | Reference |
| Yes | 965 | 73.0 (70.1–75.7) | 1.0 (1.0–1.1) | 1.1 (1.0–1.1) |

95% Confidence intervals (CI) and sample sizes (n) are provided. An asterisk (*) indicates deviations in sample sizes from the total number of 2,139 participants due to missing data.
