# Supplementary material for: Human papillomavirus awareness and vaccination willingness among adults in Madagascar: a cross-sectional study
Source: BMC Womens Health. 2025 Dec 3;25:596. doi: 10.1186/s12905-025-04199-9 (PMC12706918; doi:10.1186/s12905-025-04199-9)
Supplement: Supplementary file 6 — Supplementary Material 6. [file 12905_2025_4199_MOESM6_ESM.docx]

**Supplementary Table S5.** Prevalence, crude (cPR) and adjusted prevalence ratios (aPR) for HPV vaccination willingness for one’s son, adjusted for HPV awareness, sociodemographic and healthcare-related factors (Poisson regression analysis).

|  | **n** | **Prevalence % (95% CI)** | **Crude PR**  **(95% CI)** | **Adjusted PR (95% CI)** |
| --- | --- | --- | --- | --- |
| **Total** | 2,130 | 71.1 (69.1-73.0) | - | - |
| **HPV awareness (n = 2,131*)** |  |  |  |  |
| HPV unaware | 2,035 | 70.6 (68.6–72.6) | Reference | Reference |
| HPV aware | 96 | 81.2 (72.3–87.8) | 1.2 (1.0–1.3) | 1.2 (1.1–1.3) |
| **Region (n = 2,131*)** |  |  |  |  |
| Boeny | 1,031 | 80.9 (78.4–83.2) | Reference | Reference |
| Matsiatra Ambony | 1,100 | 61.9 (59.0–64.7) | 0.8 (0.7–0.8) | 0.8 (0.7–0.8) |
| **Urbanicity (n = 2,131*)** |  |  |  |  |
| Rural | 1,081 | 75.4 (72.7–77.9) | Reference | Reference |
| Urban | 1,050 | 66.7 (63.8–69.5) | 0.9 (0.8–0.9) | 0.9 (0.9–1.0) |
| **Sex (n = 2,131*)** |  |  |  |  |
| Male | 878 | 71.4 (68.3–74.3) | Reference | Reference |
| Female | 1,253 | 70.9 (68.3–73.3) | 1.0 (0.9–1.0) | 1.0 (0.9–1.1) |
| **Age group (n = 2,131*)** |  |  |  |  |
| 18-19 | 256 | 77.0 (71.4–81.7) | Reference | Reference |
| 20-29 | 802 | 73.7 (70.5–76.6) | 1.0 (0.9–1.0) | 1.0 (0.9–1.1) |
| 30-39 | 369 | 66.7 (61.7–71.3) | 0.9 (0.8–1.0) | 0.9 (0.8–1.0) |
| ≥40 | 704 | 68.3 (64.8–71.7) | 0.9 (0.8–1.0) | 0.9 (0.8–0.9) |
| **Education (n = 2,130*)** |  |  |  |  |
| No/primary school | 659 | 78.0 (74.7–81.0) | Reference | Reference |
| Secondary school | 1,049 | 70.4 (67.5–73.0) | 0.9 (0.9–1.0) | 0.9 (0.8–0.9) |
| Higher education | 422 | 62.1 (57.4–66.6) | 0.8 (0.7–0.9) | 0.8 (0.7–0.9) |
| **Occupation (n = 2,128*)** |  |  |  |  |
| Working | 1,630 | 71.3 (69.0–73.4) | Reference | Reference |
| Unemployed/retired | 163 | 72.4 (65.1–78.7) | 1.0 (0.9–1.1) | 1.0 (0.9–1.1) |
| Student | 335 | 70.1 (65.0–74.8) | 1.0 (0.9–1.1) | 1.0 (1.0–1.2) |
| **Contact to healthcare**  **within the last year**  **(n = 2,125*)** |  |  |  |  |
| No | 1,162 | 70.5 (67.8–73.0) | Reference | Reference |
| Yes | 963 | 72.0 (69.0–74.7) | 1.0 (1.0–1.1) | 1.1 (1.0–1.1) |

95% Confidence intervals (CI) and sample sizes (n) are provided. An asterisk (*) indicates deviations in sample sizes from the total number of 2,139 participants due to missing data.
